# Early Childhood Caries in 4- to 5-Year-Old Children in Erzurum, Turkey

**DOI:** 10.3389/fpubh.2021.725501

**Published:** 2021-11-26

**Authors:** Fatih Şengül, Gelengül Urvasızoğlu, Sera Derelioǧlu, Tarek Seddik, Periş Çelikel, Aybike Baş

**Affiliations:** ^1^Department of Pediatric Dentistry, Faculty of Dentistry, Atatürk University, Erzurum, Turkey; ^2^Department of Oral and Maxillofacial Surgery, Faculty of Dentistry, Atatürk University, Erzurum, Turkey

**Keywords:** early childhood caries, deft, SiC, SiC10, restorative index, treatment needs, care index, children

## Abstract

**Introduction:** Early childhood caries is tooth decay seen in children under 72 months old. It is associated with multiple predisposing factors and has a negative impact on quality of life. In this study, our aim was to assess the oral health conditions and prevalence of early childhood caries (ECC) in children in the city of Erzurum, Turkey.

**Materials and Methods:** This cross-sectional epidemiological study was conducted in Atatürk University, Faculty of Dentistry, Pediatric Dentistry Department/Erzurum-Turkey, in the 2015–2016 academic year. A total of 1,156 children (588 girls and 568 boys), with mean age of 4.9 ± 0.3 years (min 4, max 5) were included in the study. Restorative index (RI), deft, significant caries index (SiC), SiC10, treatment needs, number of lost primary teeth per 100 children, care index, and prevalence of carious primary teeth were evaluated. Data were analyzed by Chi-square test and Mann–Whitney U test (*p* < 0.05).

**Results:** A total of 73.3% prevalence of ECC was observed in preschool children with a mean deft score of 3.9±4 and an increase in ECC with age. RI was 2.2%, SiC was 8.5, SiC10 was 12.3, caries treatment needs was 93.5%, care index was 2.1%, and number of lost primary teeth per 100 children was 0.9 tooth.

**Conclusion:** High level of ECC indicates the necessity of starting an oral health education program for mothers and dental screening of children, and the demand for improving oral and dental services.

## Introduction

Early childhood caries is defined as the presence of one or more decayed, missing, or filled tooth surfaces in any primary tooth of children under 72 months old (1). Recently, early childhood caries (ECC) has increased rapidly worldwide, becoming an important public health problem especially in underdeveloped and developing societies. The great impact of ECC on the quality of life of children due to early tooth loss, malnutrition, and delayed growth and development, has been demonstrated in many studies (2–4). Moreover, efforts to prevent ECC have not achieved satisfactory results. Results of health surveys conducted in Turkey on 0- to 6-year-old children in 2010, 2015, and 2019 showed that 7.8, 9.3, and 6.4%, respectively, of them had oral and dental health problems (5–7). Recent oral health training and fluoride application campaign launched by Turkish Ministry of Health caused a slowdown in the increasing rate of oral and dental health problems. This study includes data from the first year of the campaign of the Ministry of Health.

In societies regarded as developing countries, such as Turkey, ECC is common because of the presence of various predisposing factors such as misconceptions about infant feeding, non-nutritive suckling habits, obesity, poor oral hygiene of mother and child, educational and socioeconomic status of the family, insufficient fluoride intake, and insufficient parental knowledge of the oral hygiene of infants (8–12). In two epidemiological studies conducted after 2010 in two different cities in Turkey (Izmir in the West and Erzurum in the East) with different sociodemographic structures, the prevalence of caries at the age of 6 was 44.8 and 76.6%, respectively (13, 14).

In 2014, the Ministry of Health in Turkey started general oral health screening programs in kindergartens and primary schools in an effort to prevent dental caries, which is considered as a widespread public health problem in Turkey. During these programs, visual presentations for oral hygiene training were followed by the application of fluoride varnishes.

Data in this study were obtained from the above-mentioned oral health screening conducted in Erzurum city by the Ministry of Health in cooperation with the Faculty of Dentistry of Atatürk University. In this study, we aimed to assess ECC prevalence in children in the city of Erzurum, Turkey, as well as the prevalence of other oral health conditions.

## Materials and Methods

This study was conducted by the Department of Pediatric Dentistry, Faculty of Dentistry, Atatürk University in accordance with the provisions of Ministry of Health Clinical Research Regulations, and had written approval from the Faculty of Medicine Research Ethics Committee (session No.03/2021 resolution # 31). In this descriptive cross-sectional study, we evaluated archived data from the oral health screening project, which was jointly carried out by the Faculty of Dentistry, Atatürk University, and the Ministry of Health, in the 2015–2016 academic year. The archived data covered 19,807 preschool and primary school children aged between 4 and 12 years in all 263 schools in Erzurum city. According to Turkish Statistical Institute (TUIK), a total of 26,866 children aged 4–12 years old were living in Erzurum around the time the data were collected; among these children, all preschoolers were included in our study (15).

### Inclusion Criteria

Day care center, kindergarten, and first-grade children under the age of 72 months were included in this study; their data were extracted from the entire database of the oral health screening program; other children were excluded. The study included children attending public and private schools, representing students from low, middle, and high-income families.

### Data Collection Tool

Study data were collected with examination forms developed by the researchers. The examination forms were prepared in order to record the dental caries status of the participants.

### Researchers

This study was carried out by three faculty members from the department of pediatric dentistry and one member from the oral and maxillofacial surgery department.

Before beginning the study, examiners were trained and evaluated at the Department of Pediatric Dentistry, Faculty of Dentistry, University of Atatürk by one of the authors (SD), who is an experienced pediatric dentist. Kappa statistic was used to compare intra- and inter-examiner agreements of the measured deft index scores in 40 children. Kappa statistic values, comparing the deft scores measured by each of the three examiners to the deft scores measured by SD, were 0.93, 0.74, and 0.91. Intra-examiner reliability, assessed in the same children with 10-day intervals, was high, and the Kappa statistic score was 0.95, 0.82, and 0.94 for the three examiners.

Following visual presentations on oral and dental health given at the schools of the participants, oral examinations of the children were performed, and results were recorded. Parents were informed about the treatment needs of their children with formal letters delivered by post. After the oral examination and recording process had been completed, varnishes containing 5% sodium fluoride (NaF) were applied to the teeth of the children.

### Oral Examination

School administrations were notified that children should brush their teeth prior to the examination. The examinations were performed in school classrooms using flat mouth mirrors, WHO periodontal probes, dental gauze rolls, containers for dirty tools, rubber gloves, single-use surgical masks, hand sanitizers, paper towels, and pen lights. First, the examining dentists explained the examination procedure to children one by one. Then, the children were examined seated in a high-back seat facing the examining dentist.

### Study Size

The study population included a total of 1,156 children (568 boys and 588 girls), 4–5 years old, in the city of Erzurum. Sample size was calculated as 1,112 using Epi Info^TM^6 (with 99% confidence interval, 5% standard error, and 73.3% prevalence). We tried to reach the whole population, and 1,156 children whose families were willing to participate in the study were included. Students who were absent on the day of the oral examination (*n* = 57) were evaluated in their schools the following week. Thus, this research can be generalized to children aged 4–5 years living in Erzurum.

### Indices Used

In this study, the following indices were evaluated: caries prevalence in primary teeth, number of decayed, extracted due to caries, or filled teeth (deft), significant caries index (SiC, the mean deft of 1/3 of the study group with the highest caries score), SiC10 index (the mean deft of children with 10% highest deft scores), number of missing (extracted) primary teeth per 100 children (the sum of extracted primary teeth divided by the number of children and multiplied by 100), $caries\;treatment\;needs=\frac{decayed\;teeth}{deft}\times 100\%$, $care\;index=\frac{filled\;teeth}{deft}\times 100\%$, and restorative index (RI $=\frac{decayed\;teeth}{decayed\;teeth+filled\;teeth}\times 100\%$) (16, 17).

The number of missing primary teeth per 100 children was modified from another index (number of missing permanent teeth per 100 children) that was previously used for permanent dentition (18).

### Statistical Analysis

All statistical evaluations were carried out using Statistical Package for Social Sciences version 26 (SPSS Inc., Chicago IL, United States). Descriptive statistics were used to determine the sociodemographic and clinical status of included children. Kolmogorov–Smirnov test indicated that the distribution of deft and index scores did not follow a normal distribution. Therefore, Pearson Chi-square test (nominal data) was performed for enumeration data, and Mann–Whitney U test (two groups) was performed for continuous data. The level of statistical significance was set at *p* < 0.05.

## Results

Out of the 1,156 research participants, 568 (49.1%) were boys and 588 (50.9%) were girls, with a mean age of 4.9 ± 0.3 years. A homogeneous distribution was observed between the groups in terms of age and sex (*p* = 0.303).

The prevalence of dental decay was 73.3% (Table 1). Total study population mean deft score was 3.9 ± 4. SiC score for the total population was 8.5, and SiC10 score was 12.3. Caries prevalence, deft, SiC, and SiC10 scores of 4-year-old children were lower than those of 5-year-olds (*p* < 0.05). Although there was no significant difference in caries prevalence between the sexes (*p* > 0.05), deft, SiC, and SiC10 scores of the girls were significantly lower than those of the boys (*p* < 0.05).

**Table 1:** Deciduous caries status of different age and sex (*n* = 1,156).

**Variables**	**Categories**	**Caries prevalence**			**deft**		**SiC**		**SiC10**	
		**no. of surveyed (No. of cases with dental caries)**	**Caries prevalence (%)**	**p-value (X^**2**^ test)**	**mean ± SD**	**p-value (Mann-Whitney U)**	**mean ± SD**	**p-value (Mann-Whitney U)**	**mean ± SD**	**p-value (Mann-Whitney U)**
Age (year)	4	91 (49)	53.8	<0.001	2.1 ± 3	<0.001	5.4 ± 3.2	<0.001	9.6 ± 2.2	0.002
	5	1065 (768)	74.9		4 ± 4		8.8 ± 3		12.5 ± 2.3	
Sex	Boys	588 (424)	72.1	0.364	4.2 ± 4.2	0.014	9.2 ± 3.1	<0.001	13.1 ± 2.6	0.002
	Girls	568 (423)	74.5		3.6 ± 3.7		8 ± 2.8		11.6 ± 1.7	
Total	-	1156 (847)	73.3	-	3.9 ± 4	-	8.5 ± 3.1	-	12.3 ± 2.3	-

*deft, the number of decayed, extracted due to caries, or filled teeth; SiC, significant caries index; SiC10, significant caries index of the 10% of children with the highest deft scores; SD, standard deviation*.

**Figure 1:**
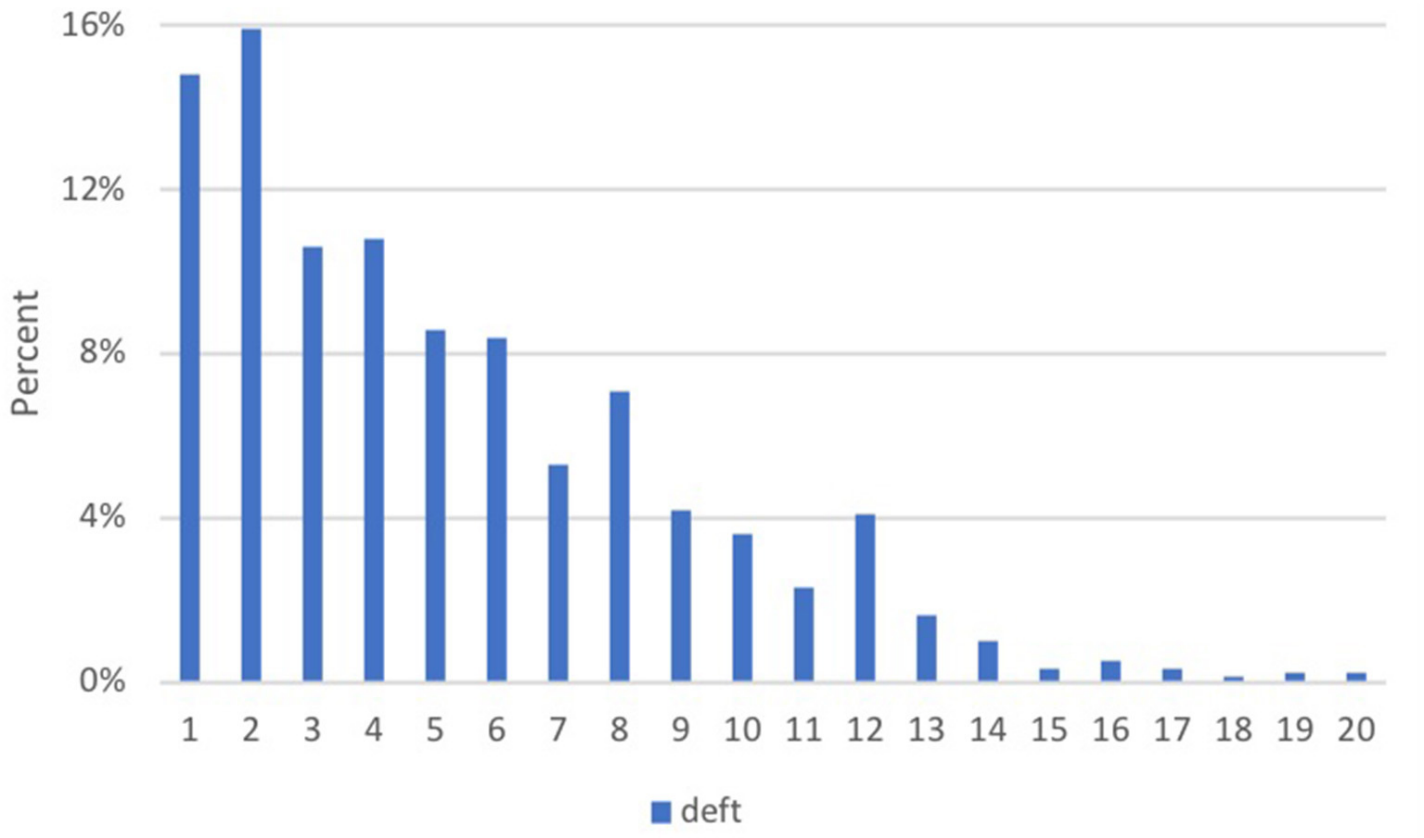
Frequency distribution of the number of decayed, extracted due to caries, or filled teeth (deft) in 1,156 children aged 4 to 5 in Erzurum community in Turkey for the 2015–2016 academic year.

The distribution of deft frequency is given in Figure 1 (“deft = 0” score was excluded). The most frequent deft values were 2, 1, and 4 scores (15.9, 14.8, and 10.8%, respectively). The distribution of the number of carious primary teeth per 100 children, caries treatment needs, care index, and RI according to age and sex is illustrated in Table 2. There were no significant differences between age and sex in these indices (*p* > 0.05).

**Table 2:** The distribution of different indices of 1,156 children by age and sex.

**Age (year)**	**Sex**	**Carious primary teeth per 100 children (teeth)**	**Caries treatment needs (%)**	**Care index (%)**	**RI (%)**
4	Boys	0.8	91.7	2.1	2.2
	Girls	0.1	97.9	1	1.1
5	Boys	1	93.2	2.4	2.5
	Girls	0.9	93.7	1.9	2
Total		0.9	93.5	2.1	2.2
p-value (X^2^ test)	0.386	0.799	0.659	0.85

*RI, restorative index*.

## Discussion

Early childhood caries is one of the preventable chronic diseases that are progressing internationally in recent years. According to The Global Burden of Disease Study-2017, ~530 million children were estimated to have untreated primary teeth caries (19). It is a serious public health problem caused by the interaction among cariogenic bacteria, carbohydrates, improper nutrition conditions, and a number of social factors (20–22). ECC has a negative impact on the well-being, learning skills, and growth and development of children. In short, it affects their quality of life (23–25). Therefore, oral and dental health should also be evaluated within the scope of general health protection programs. Since many families cannot afford costly advanced ECC treatments conducted under general anesthesia or sedation, ECC also adversely affects the families by imposing a heavy economic burden (26). This burden is huge, and immediate measures are needed to promote the oral health of children worldwide. Providing the community with oral healthcare services could be achieved with government policies; these policies may address several structural factors predisposing to ECC. Countries have developed their own plans and projects to intervene against ECC (27).

Because of increasing demand for dental treatments in pediatric patients in recent years in Turkey, the Ministry of Health launched countrywide oral and dental health screening programs in 2014. Within the scope of this program, not only condition assessment was made, but also educational seminars for children and teachers on oral hygiene measures and caries prevention were organized, and 5% NaF varnishes were applied to children. This study covered a part of this large project, which was conducted in Erzurum and its surroundings, and was jointly run by Atatürk University Faculty of Dentistry and the Ministry of Health.

This is not the first study of the researchers on ECC in the province of Erzurum. They had also performed a less comprehensive study in Erzurum in 2002 and 2013 that included similar age groups (8, 14). In this section, we will compare data from Erzurum province to data from Turkey and other countries.

Studies that evaluated caries prevalence showed that caries increased proportionally with age (28, 29). In our study, the mean value of caries prevalence was 73.3% for children aged between 4 and 5 years, raising serious concern about the status of permanent teeth in these children.

The higher scores obtained in this study, compared to our previous study which was conducted on a smaller population (63% in 4-year-old and 64.4% in 5-year-old children), (14) indicate an increase in the prevalence of ECC over time. Furthermore, we think that the results of this study are more generalizable, since it has been carried out on a larger population. Regardless, these data clearly indicate that the future of oral and general health status of these children is not promising.

According to the WHO, ECC prevalence by continents is: Africa 30%, Americas 48%, Asia 52%, Europe 43%, and Oceania 82%. Considering the 48% global ECC prevalence, the prevalence of 73% found in our study is quite high (30). However, our study is limited within the Erzurum province. According to the 2011 data from the Ministry of Development with regard to socioeconomic development status, Erzurum ranked 59th among 81 cities in Turkey. As reported in the 2017 urban development index by the Ministry of Industry and Technology, Erzurum province ranked 5th out of six levels, indicating lower urban development index (31). Hence, we believe that the high prevalence of ECC found in our study is linked to low-income levels.

The ratio of available dental workforce to dental caries prevalence makes it unrealistic to suggest providing surgical or conventional restorative treatments to millions of children suffering from ECC in low-income countries. New intervention protocols that are highly effective, easy to deliver, and require fewer sensitive techniques and dental equipment are needed, especially in countries with high ECC prevalence and low dentist/population ratios.

In such low income countries, taking into consideration the lack of dental workforce and high ECC prevalence, recommending the provision of conventional dental treatment to millions of children is impractical. Novel and effective treatment protocols that can be easily fulfilled with less equipment and simple methods are required in countries with high ECC prevalence and low dentist/population ratios (32). Similar to the situation remarked by Chen et al. (32) in their review, only 6 pediatric dentists and 12 research assistants have led the efforts against ECC in Erzurum where the pediatric dentist/population ratio is 1/11,300 for children aged 0–14 years.

In our study, higher index values found in 4- to 5-year-old children, with caries treatment needs value of 93.5%, deft score of 3.9, and SiC score of 8.5, revealed that caries-preventive measures for this population were insufficient. In a study carried out on 552 kindergarten children with mean age of 4.4 ± 0.7 years in Bucharest, Romania, the deft and SiC indices were 9.11 and 12.6, respectively (33). Although this study had a higher mean score for age, which was 4.9 years, our deft score was lower but with a similar SiC score.

Mothupi et al. (34) reported caries prevalence of 48.7%, deft and SiC indices of 2.4 and 6.4, respectively, in children aged between 4 and 5 years in South Africa. In another study, Hoffmeister et al. (35) reported a deft score of 2.5 and a SiC value of 6 for 4-year-old children in Chile. Those in our findings were higher than these values.

With the help of results obtained in our study, the need for implementation of minimally invasive approaches, such as atraumatic restorative treatment, and non-surgical approaches, such as the Hall Technique and silver diamine fluoride application, and the need for educating the public on ECC, including how to improve nutrition status and oral hygiene, were emphasized once more (36). The high prevalence of caries in our study indicates the indifference of our society to oral health of infants and young children. Moreover, although efforts were made to prevent ECC by the Ministry of Health, such as semiannual dental field screenings, fluoride varnish applications, and distribution of toothpastes and toothbrushes, measures for tackling ECC are still insufficient. Children with very high prevalence of caries needed comprehensive dental rehabilitation.

## Conclusion

This study evaluated 4- to 5-year-old children living in Erzurum, Turkey, regardless of their socioeconomic status and nutritional habits, and found very high caries prevalence. There will be a need for future studies in order to evaluate risk factors, such as socioeconomic status and nutritional and oral hygiene habits, that increase the prevalence of caries in our population.

## Data Availability Statement

The original contributions presented in the study are included in the article/Supplementary Material, further inquiries can be directed to the corresponding author.

## Ethics Statement

This study was conducted by the Department of Pediatric Dentistry, Faculty of Dentistry, Atatürk University in regard to the provisions of Ministry of Health Clinical Researches Regulations and also in accordance with the Faculty of Medicine Research Ethics Committee's written approval (Session No. 03/2021 resolution # 31). Written informed consent to participate in this study was provided by the participants' legal guardian/next of kin.

## Author Contributions

FŞ and GU: scanning archive data, examination of patients, and manuscript preparation. SD: study design, scanning archive data, and manuscript preparation. TS: examination of patients and manuscript preparation. PÇ and AB: statistical analyses and manuscript preparation. All authors contributed to the article and approved the submitted version.

## Conflict of Interest

The authors declare that the research was conducted in the absence of any commercial or financial relationships that could be construed as a potential conflict of interest.

## Publisher's Note

All claims expressed in this article are solely those of the authors and do not necessarily represent those of their affiliated organizations, or those of the publisher, the editors and the reviewers. Any product that may be evaluated in this article, or claim that may be made by its manufacturer, is not guaranteed or endorsed by the publisher.
